# Extension of Prophylactic Surgery in Medullary Thyroid Carcinoma. Differences Between Sporadic and Hereditary Tumours According to Calcitonin Levels and Lymph Node Involvement

**DOI:** 10.1007/s00268-022-06448-6

**Published:** 2022-01-28

**Authors:** L. D. Juez, E. Mercader, I. Amunategui, B. Febrero, J. M. Rodríguez, J. Gómez-Ramírez, MECANO Collaboration Group, R. Arranz Jimenez, F. García Lorenzo, M. Domínguez, L. Lorente, J. Girones, A. Mira, A. De la Quintana, J. L. de Nova Muñoz, I. Osorio, A. Carrión, O. González, M. Beisani, R. San Román, N. Muñoz, J. Bonnin, C. Alvarez Segurado, M. Jiménez Segovia, J. César Jordán, X. Girao, M. F. Candel Arenas, L. Gallego, C. Fernández, A. Camacho, C. Jiménez, I. Gutierrez, D. Acín, M. Darriba, M. L. de Molina Sánchez, M. A. Vaquero, P. Garaulet

**Affiliations:** 1 Department of General Surgery, Hospital Universitario Ramón y Cajal, Ctra. Colmenar Viejo, Km 9.100., 28034 Madrid, Spain; 2 Department of General Surgery, Division of Endocrine Surgery, Hospital Universitario Gregorio Marañon, Madrid, Spain; 3 Department of General Surgery, Division of Endocrine Surgery, Hospital Universitario Virgen de La Arrixaca, Murcia, Spain

## Abstract

**Introduction:**

Currently, there is no consensus on the indication of prophylactic surgery of the nodal compartments in the treatment of medullary thyroid carcinoma (MTC). The aim of our study was to perform a correlation study between preoperative calcitonin (basalCT) values and lymph node involvement to establish a criterion on which to base prophylactic surgery in these patients.

**Material and Methods:**

We conducted an observational, retrospective and multicentre study with 29 hospitals. Patients over 18 years of age with a diagnosis of MTC with a pre-surgical calcitonin registry were included. The minimum surgery in all patients had to have been total thyroidectomy (TT) with central compartment lymph node dissection (CCLND). Receiver operating characteristic (ROC) curve analysis was used to establish basalCT cut-off values as predictors of postoperative lymph node involvement.

**Results:**

A total of 244 patients were included. Baseline calcitonin (basalCT) was a good predictor of nodal involvement (AUC 0.718 and 95%CI 0.66–0.978). Heritability was identified as a preoperative factor correlated with baseline tumour CT values (*p* = 0.000). With a probability of lymph node involvement below 10%, new cut-off points were established. A prophylactic bilateral lateral lymph node dissection in sporadic tumours should be performed at a basalCT > 600 pg/mL; in the case of RET-mutated tumours this value would be 200 pg/mL.

**Conclusion:**

The baseline CT value is a good predictor of postoperative lymph node involvement in MTC, however, cut-off points should depent on the hereditary nature of the tumour.

## Introduction

Medullary thyroid carcinoma (MTC) is the third leading cause of malignant thyroid neoplasms [1]; however, it has declined in recent years and currently represents approximately 1–2% of thyroid cancer diagnoses [2].

There are several aspects that differentiate MTC from other thyroid neoplasms: (1) MTC originates in the malignization of parafollicular cells (C cells); the therapeutic arsenal against MTC is therefore limited; (2) MTC generally progresses more aggressively with greater recurrence and mortality; (3) MTC is multicentric in 90% of hereditary tumours and in 20% of sporadic tumours; and (4) MTC has a specific marker: calcitonin [3–8].

Numerous studies have linked preoperative calcitonin levels with lymph node involvement in MTC [9–14]. Although most authors accept that total thyroidectomy (TT) with central compartment lymph node dissection (CCLND) should be the minimal surgery for these patients [2, 15, 16], there is no uniform recommendation on the extent of prophylactic lateral lymph node dissections (LLND). There are authors who recommend performing a prophylactic lymphadenectomy according to calcitonin levels [2, 9–11, 17]. Due to this procedure’s potential morbidity [18–20], other authors recommend limiting the extent of lymphatic dissection to territories where pathological lymph nodes are detected by preoperative ultrasound [2].

Due to the disparity in the criteria for managing nodal disease in MTC, there are still no standard recommendations for managing the disease [15, 16]. Moreover, no previous study on the subject has made a distinction between hereditary and sporadic disease, instead assuming that the tumour’s biological behaviour is similar in both cases.

Our objective was therefore to perform a correlation study between preoperative calcitonin values and lymph node involvement to establish concrete criteria on which to base prophylactic surgery for these patients.

## Material and methods

### Design

We conducted an observational, retrospective and multicentre study based on each centre’s prospective medical records in a common, computerised and anonymised database to which only the study’s principal investigator had access. Participation was offered to the members of the endocrine surgery section of the Spanish Association of Surgeons, and 29 centres were ultimately recruited.

### Inclusion criteria

The inclusion criteria were patients older than 18 years with a diagnosis of MTC who underwent surgery between January 2000 and July 2020 and had a record of their preoperative calcitonin level. The minimal surgery for all patients should have been TT and CCLND. Depending on the degree of suspicion and each centre’s protocol, ipsilateral or contralateral LLND was performed. We defined the lymph node dissection as appropriate for study inclusion when it included at least 4 lymph nodes in the CCLND and 10 lymph nodes in the LLND in the pathology review of the surgical specimens.

The exclusion criteria were incomplete lymphadenectomies, the absence of postoperative follow-up and the presence of preoperatively confirmed metastatic disease.

### Variables

We collected the following preoperative information: demographic (sex and age), hereditary nature of the tumour, preoperative laboratory values (preoperative [basalCT] and postoperative calcitonin levels [postCT] in pg/mL), preoperative and postoperative carcinoembryonic antigen (CEA) levels (basalCEA or postCEA in ng/mL) and the presence of preoperative lymph node involvement in the neck ultrasound.

We included the following postoperative variables: tumour stage based on the TNM classification according to the 8th edition of the American Joint Committee on Cancer staging system [21], surgical technique (TT with CCLND, TT with CCLND and ipsilateral LLND and TT with CCLND and bilateral LLND), tumour size (in mm) and number of lymph nodes in each compartment and their involvement.

### Disease persistence

We classified postoperative disease persistence into 3 groups depending on the laboratory results at 3–6 months after the surgery [15]: *excellent response or healed* if the postCT was < 20 pg/mL, *incomplete biochemical response* if the postCT was > 20 pg/mL with no evidence of structural disease, and *structural persistence* if there was radiological/histological/cytological confirmation of disease, regardless of postCT levels.

### Disease recurrence

We considered disease recurrence if there was an increase in CT or evidence of structural disease in the patients previously classified as having an excellent response or healed*.*

### Statistical analysis

We checked the normality of the continuous variables using the Kolmogorov–Smirnov nonparametric test. The quantitative variables are presented with the mean and standard deviation values if the distribution was normal and with the median and interquartile range (IQR) values otherwise. The categorical variables are expressed as absolute numbers and percentages.

For the statistical analysis of the quantitative variables, we employed Student’s t-test. For non-normal variables, we used nonparametric tests, and for the categorical variables, we applied Pearson’s chi-squared test.

To identify any possible subgroups in the baseline characteristics of the samples associated with basalCT levels, we used Spearman’s correlation coefficient.

To establish the preoperative calcitonin cut-off values as predictors of postoperative lymph node involvement, we employed an analysis of receiver operating characteristic (ROC) curves. We also calculated the area under the curve (AUC) and its standard error.

For the selected cut-off points, we calculated the indices of diagnostic reliability: sensitivity, specificity, positive predictive value and negative predictive value. We considered a *p*-value < 0.05 statistically significant, and all ranges were calculated for a 95% confidence interval.

For the statistical analysis, we employed the statistical package SPSS statistical software version 23.0 (SPSS Inc., Chicago, IL, USA).

### Ethics committee

The study was approved by the Medical Research Ethics Committee of the University Hospital Ramon y Cajal (registration number 348/20). All of the procedures performed with human participants conformed to the research committee’s ethical standards and to the 1964 Declaration of HELSINKI and its subsequent amendments and comparable ethical standards.

## Results

**Fig. 1:**
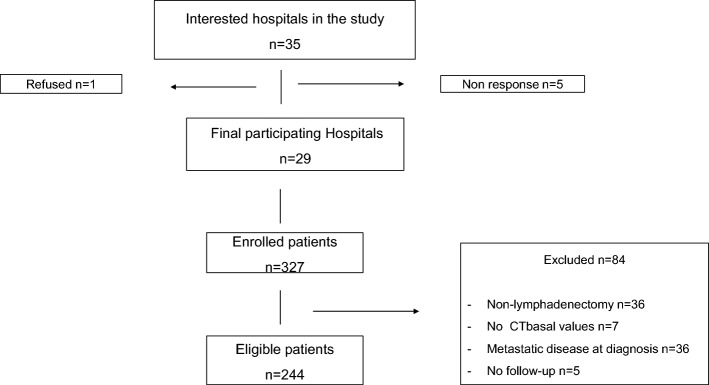
Flow diagram for study participants

Initially, 35 hospitals showed interest; however, 5 of them did not refer patients, and 1 subsequently declined to participate. Ultimately, 29 hospitals referred 327 patients. We excluded 84 patients (36 patients for incomplete lymphadenectomies, 7 due to lack of basalCT, 36 for metastasis at diagnosis and 5 due to lack of subsequent follow-up) and ultimately analysed 244 patients who met the inclusion criteria (Fig. 1).

Table 1 shows the sample’s baseline characteristics. The mean age at diagnosis was 54.9 ± 16 years, and there was a predominance of women (59%; 144 patients). Mutations associated with hereditary MTC (RET gene mutation) were not identified in 69% (168 patients), and the median basalCT was 486 (175–1550) pg/mL. The surgery most often performed was TT with CCLND (34.5%; 84 patients) followed by TT + CCLND + bilateral LLND (33%; 81 patients).

**Table 1:** The sample’s baseline characteristics

Values	*n* = 244
Female sex	59% (144)
Age (years)	54.9 ± 16
Sporadic type	69% (168)
Tumour diametre (mm)	19 (11–30)
Basal Calcitonin value (pg/mL)	486 (175–1550)
Basal CEA value (ng/mL)	21 (6–74)
Lymph nodes Ultrasound	41% (102/244)
Central compartment	10% (10/244)
Lateral compartment	90% (92/244)
Extra-thyroidal extension	10% (25/244)
TNM stage -8th edition AJCC^a^	
I	32% (78)
II	15.6% (38)
III	19% (46)
IVA	31% (76)
IVB	2% (5)
Surgery	
TT^b^ + CCLND^c^	34.5% (84)
TT^b^ + CCLND^c^ + LLND ipsilateral^d^	32% (78)
TT^b^ + CCLND^3^ + LLND^d^ bilateral	33% (81)
Others	0.5% (1)
Tumour size (mm)	17 (11–30)
Multifocality	39% (95)
Lymph node dissection	
CCLND	
Positive	125/240 (52%)
Dissected lymph nodes (median)	7 (5–11)
Positive dissected lymph nodes (median)	1 (0–3)
LLND ipsilateral^d^	
Positive	104/157 (66%)
Dissected lymph nodes (median)	16 (12–23)
Positive dissected lymph nodes (median)	2 (0–6)
LLND contralateral^d^	
Positive	31/78 (40%)
Dissected lymph nodes (median)	13 (8–21)
Positive dissected lymph nodes (median)	0 (0–3)
Postoperative Calcitonin value (pg/mL)	5.6 (0–65)
Postoperative CEA value (ng/mL)	2 (1–4)
Persistence	
Biochemical	65/244 (26%)
Structural	14/244 (5.7%)
Recurrence	
Total	25/165 (15%)
Structural	23
Biochemistry	2
All-cause mortality	33 (13.6%)
Cancer-specific mortality in medullary thyroid cancer	29 (11.9%)
Follow-up (months)	63 (32–140)

^a^8th edition of Tumor, lymph Nodes and Metastasis staging system by The American Joint Committee on Cancer

^b^Total thyroidectomy

^c^Central compartment lymph node dissection

^d^Lateral lymph node dissections

In terms of CCLND, the median number of lymph nodes excised was 7 (5–11) nodes, with a median of 1 (0–3) lymph nodes affected. In the LLND ipsilateral to the tumour, a median of 16 (11.75–23) lymph nodes were extracted, with a median of 2 (0–6) lymph nodes affected. In the contralateral lateral compartment to the tumour, the median number of total lymph nodes was 13 (8–21), with involvement of 0 (0–2.75) lymph nodes.

The median postCT was 5.6 (0–64.5) pg/mL. We identified biochemical persistence in 65 (26%) of the patients and structural persistence in 14 (5.7%) of the patients. The recurrence rate in general (CT elevation or evidence of structural disease in patients previously classified as having an excellent response or healed) was 15% (25 patients); in most cases, the recurrence was structural (23 patients). With a median postoperative follow-up of 63 (31.7–139.7) months, we identified 33 deaths, 29 of which were cancer-specific.

To assess a factor at diagnosis as a predictor of postoperative lymph node involvement, we used the analysis based on ROC curves.

**Fig. 2:**
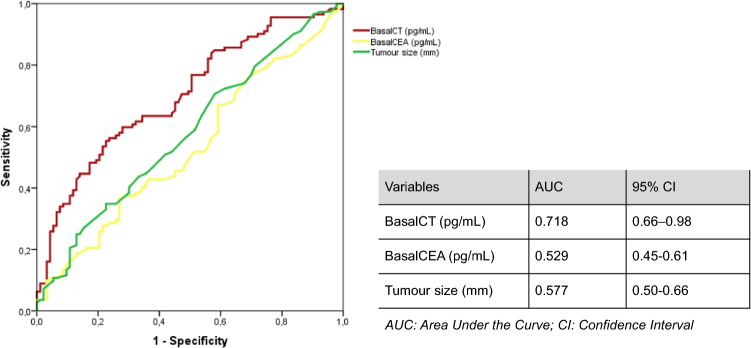
ROC curve of the basalCT, basalCEA and tumour size and lymph node involvement

The ROC curve of the basalCT and lymph node involvement in any territory showed an AUC of 0.718 and a 95% confidence interval (CI) of 0.66–0.978. In addition, the ROC curve of the pre-surgical tumour size measured by ultrasound (in mm) and the ROC curve of the basalCEA value were calculated; the results are shown in Fig. 2.

We performed a correlation analysis to evaluate the relationship between presurgical factors and basalCT values. We identified the germline RET mutation as a factor associated with basalCT values (*p* = 0.000; Spearman’s rank correlation coefficient − 0.248) (Table 2).

**Table 2:** Correlation between basalCT values and patient characteristics

	Correlation coefficient^a^	*p*
Age (years)	0.071	0.267
Gender	0.106	0.100
RET gen mutation	**− 0.248**	**0.000**

Statistical test used

^a^Spearman’s rank correlation coefficient

**Fig. 3:**
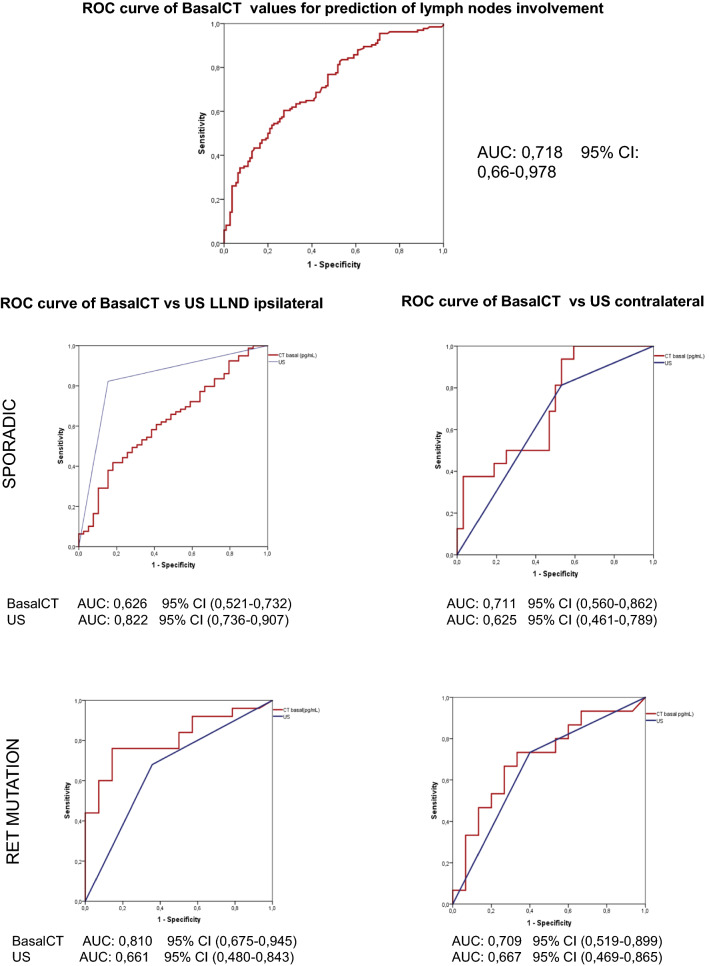
Relationship between BasalCT and lymph node involvement by lymph node regions, as well as pre-surgical ultrasound

To establish the relationship between basalCT and lymph node involvement by lymphatic territory, and as to compare these results with the preoperative ultrasound suspicion, we constructed separate ROC curves. Similarly, we divided the results by the hereditary nature of MTC. In the sporadic tumours, the basalCT AUC in the ipsilateral lateral compartment to the tumour was 0.626 (95% CI 0.521–0.732) compared with the ultrasound suspicion, which obtained an AUC of 0.822 (95% CI 0.736–0.907). In the contralateral compartment to the tumour, the data were in favour of the basalCT, with an AUC of 0.711 (95% CI 0.560–0.862). For the hereditary tumours, all of the results were in favour of the basalCT, with an AUC of 0.810 (95% CI 0.675–0.945) and an AUC of 0.709 (95% CI 0.519–0.899) in the lateral and contralateral compartments to the tumour, respectively. Figure 3 shows the results in the form of a graph.

**Fig. 4:**
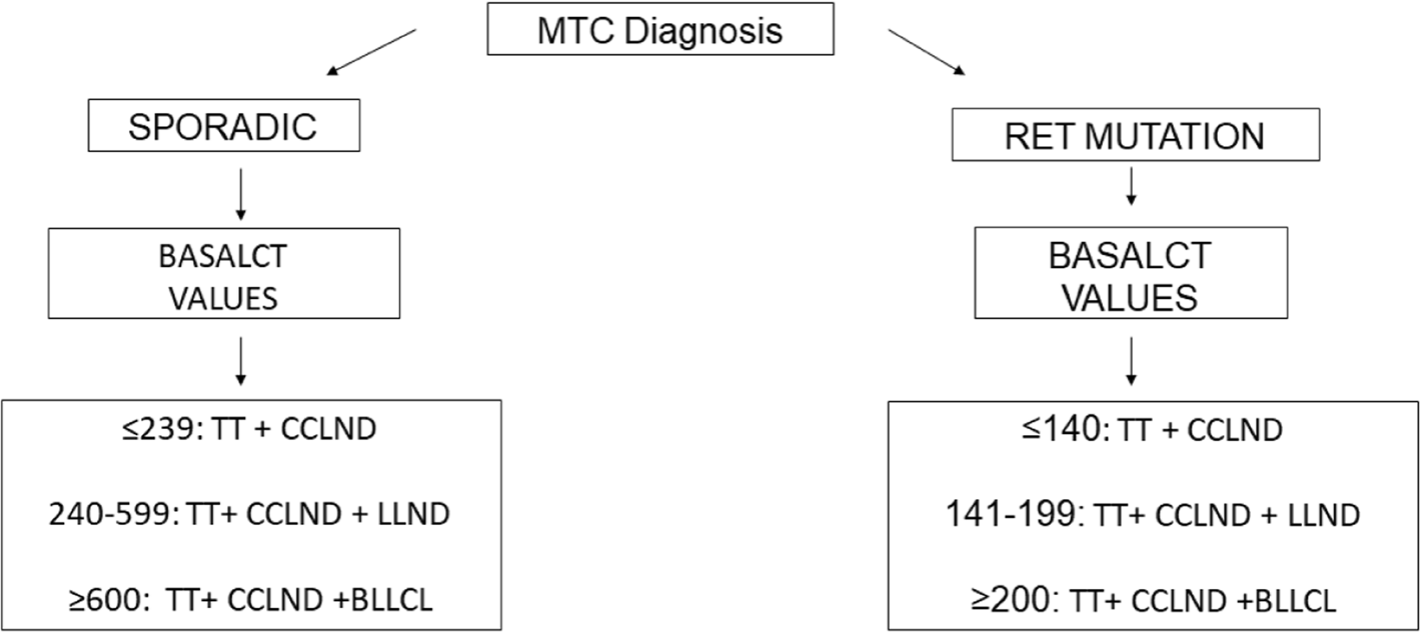
A new proposal algorithm for managing MTC based on its sporadic or hereditary nature according to basalCT levels

With the results of the ROC curves, we established the basalCT cut-off points with a sensitivity of 87–90% for detecting postoperative lymph node involvement. We established the best cut-off points in general and separately by compartment and tumour inheritability (Table 3). We constructed an algorithm for managing prophylactic MTC node dissection based on the heritable nature of the tumour and baseline CT values (Fig. 4). After the MTC diagnosis, no detection of heritable genetic mutations and no detection of pathological nodes, if basalCT levels are < 240 pg/mL, a TT + CCLND will be performed. In case of basalCT levels of 240–599 pg/mL, TT + CCLND and LLND will be performed. In case of basalCT levels >600 pg/mL, the recommendation is to finish with bilateral lateral cervical dissection. For hereditary tumours, the basalCT values were < 140 pg/mL, 141–199 pg/mL and >199 pg/mL, respectively.

**Table 3:** New proposed basal CT values

	All		Sporadic type		Hereditary type	
	CTbasal values	LN +^c^	CTbasal values	LN +^c^	CTbasal values	LN +^c^
CCLND^a^	171	11%	200	10%	87	10%
LLND^b^ Ipsilateral	237	11%	240	10%	142	12%
LLND^b^ contralateral	339	10%	627	12%	200	13%

^a^Central Compartment lymph node dissection

^b^Lateral lymph node dissections

^c^Lymph nodes involvement

## Discussion

The established curative treatment for MTC is surgery; specifically, the European, British and American Thyroid Associations (ATA) and the American Association of Endocrine Surgeons, recommend TT and CCLND, regardless of the state of lymph node involvement [2, 15, 16, 22, 23]. However, the extent of prophylactic lateral lymph node dissection has not been completely standardised, and there are numerous authors who recommend the baseline calcitonin level as a guide [2, 5, 11–13].

The ATA recommends performing TT with level VI lymph node dissection and completing the lateral lymph node dissection according to the impairment suggested by the preoperative ultrasound. For cases with basalCT > 200 pg/mL, the ATA suggests the option of completing the lymph node dissection in the contralateral lateral compartment [2]. Moreover, there is the European trend recommending broadening the lymph node dissection based on basalCT values. In particular, Machens et al. found that the lateral involvement was >75% with 1–3 positive central compartment lymph nodes, a percentage that increases to more than 98% with 4 or more such nodes [9], thereby confirming the metastasising ability of MTC. The authors subsequently recommended performing central compartment lymph node dissection ipsilateral and ipsilateral lateral to the tumour if the basalCT was 20–50 pg/mL, bilateral central and ipsilateral lateral lymph node dissection to the tumour if the basalCT was 50–200 pg/mL, and completing the contralateral side if the basalCT was >200 pg/mL [10, 11, 17]. In line with these results, Hyunju et al. broadened the range of basalCT values to 0–100 pg/mL in the central compartment and up to 300 pg/mL in the lateral and contralateral lateral compartment [24].

Our results follow the trend described in the literature [11, 13, 24, 25], although with less stringent cutoff points than those reported by Machens et al. The basalCT value is a good predictor of postoperative lymph node involvement in MTC (AUC 0.718, 95% CI 0.66–0.978). When we performed a subgroup analysis of our sample, we identified the tumour’s hereditary nature as a preoperative factor correlated with the tumour’s basalCT values (*p* = 0.000; Spearman’s rank correlation coefficient − 0.248).

Over the past few years, knowledge of many aspects of multiple endocrine neoplasia type 2 (MEN2) has expanded [26]. To date, however, the main guidelines on managing MTC have not independently established separate cut-off points for sporadic MTC versus hereditary MTC and have varied the aggressiveness of the surgery depending only on the specific type of tumour mutation [2, 15, 16, 23, 27, 28].

In our study’s subgroup analysis, we identified different basalCT values between sporadic tumours and tumours associated with the RET mutation. For basalCT values < 200 pg/mL in sporadic tumours and 87 pg/mL in tumours with the RET mutation, the probability of finding affected lymph nodes in the central compartment was < 10%. For basalCT values of 200–240 pg/mL in the ipsilateral lateral compartment in sporadic tumours and 87–142 pg/mL in hereditary tumours, the probability of detecting lymph node disease was 10–12%. For basalCT values of 240–627 pg/mL in sporadic tumours and 142–200 pg/mL in hereditary tumours, the probability of not having positive lymph nodes in the compartment contralateral lateral to the tumour was >87% (Table 3).

In terms of ultrasonography and according to the ATA recommendations [2], we assessed the reliability of this technique in the patients. In our sample, the AUC of ultrasonography as a prognostic test for postoperative lymph node involvement was 0.8. However, in the analysis by tumour type and lymph node compartment, the AUC of ultrasonography was only greater than the basalCT value in the ipsilateral lateral compartment in sporadic tumours (basalCT AUC 0.626, 95% CI 0.521–0.732; ultrasonography AUC 0.822, 95% CI 0.736–0.907) (Fig. 3).

Based on our results and as a completely new option, we propose an algorithm for managing MTC based on its sporadic or hereditary nature according to basalCT levels (Fig. 4), provided the preoperative ultrasound shows no clear lymph node involvement. We should first consider the tumour’s hereditary nature. In sporadic tumours and after a negative neck ultrasound, the minimal indicated surgery would be TT + CCLND. If the basalCT value is 240–600 pg/mL, the surgery would be extended with an LLND ipsilateral to the tumour. If the basalCT value is > 600 pg/mL, the surgery would be completed with the contralateral lateral compartment to the tumour.

For tumours in which the RET mutation is detected, the basalCT values will be stricter. We therefore propose TT + CCLND if the basalCT values are < 140 pg/mL, TT + CCLND + LLND if the basalCT values are 140–199 pg/mL, and TT + CCLND + bilateral LLND if the basalCT values are > 200 pg/mL (Fig. 4).

With these cut-offs, the minimum surgical act in MTC should, in our opinion, be TT with CCLND due to the high rate of central lymph node involvement and the goal of decreasing as much as possible the need for reoperations in an already operated-on compartment, regardless of the tumour’s mutational state. In the lateral neck compartments, in contrast, we assume new less strict cut-off points that are separated by the tumour’s hereditary nature. We assume a 10% rate of false negatives, which we believe is an appropriate risk, because in cases in which biochemical and/or structural persistence is detected early in the follow-up (given that these involve compartments that have not been operated on), a second operation should not entail added morbidity. Ultrasonography of the lateral compartments of the neck has a higher sensitivity and specificity than in the central compartment, which is interfered with by the thyroid glands [29, 30].

Our study has a number of limitations. This was a retrospective and multicentre study, and the results therefore have intrinsic variability related to each centre’s protocols, which we have attempted to mitigate with very strict selection criteria. We therefore consider that our data have high validity. Given the retrospective recruitment of 20 years, it is possible that each centre’s protocols could have changed over the years. Nevertheless, the surgical treatment of MTC has not experienced major changes in recent years.

## Conclusion

The baseline CT value is a good predictor of postoperative lymph node involvement in MTC, however, cut-off points should depent on the hereditary nature of the tumour.

The combination of basalCT, preoperative ultrasonography and MTC type can help in the decision-making process when deciding on the extent of lymph node dissection in MTC.

## References

[1] Melvin KE, Tashjian AH (1968). The syndrome of excessive thyrocalcitonin produced by medullary carcinoma of the thyroid. Proc Natl Acad Sci U S A.

[2] Wells SA, Asa SL, Dralle H, Elisei R, Evans DB, Gagel RF (2015). Revised American thyroid association guidelines for the management of medullary thyroid carcinoma. Thyroid.

[3] Moley JF (2010). Medullary thyroid carcinoma: management of lymph node metastases. JNCCN J Natl Compr Cancer Netw.

[4] Joaquin GR (2020) Cirugía endocrina. Cirugía Endocrina

[5] Viola D, Elisei R (2019). Management of medullary thyroid cancer. Endocrinol Metab Clin North Am.

[6] Kebebew E, Ituarte PH, Siperstein AE, Duh QY, Clark OH (2000). Medullary thyroid carcinoma: clinical characteristics, treatment, prognostic factors, and a comparison of staging systems. Cancer.

[7] Pusztaszeri MP, Bongiovanni M, Faquin WC (2014). Update on the cytologic and molecular features of medullary thyroid carcinoma. Adv Anat Pathol.

[8] Costante G, Durante C, Francis Z, Schlumberger M, Filetti S (2009). Determination of calcitonin levels in C-cell disease: clinical interest and potential pitfalls. Nat Clin Pract Endocrinol Metab.

[9] Machens A, Hauptmann S, Dralle H (2008). Prediction of lateral lymph node metastases in medullary thyroid cancer. Br J Surg.

[10] Machens A, Schneyer U, Holzhausen HJ, Dralle H (2005). Prospects of remission in medullary thyroid carcinoma according to basal calcitonin level. J Clin Endocrinol Metab.

[11] Machens A, Dralle H (2010). Biomarker-based risk stratification for previously untreated medullary thyroid cancer. J Clin Endocrinol Metab.

[12] Niederle MB, Scheuba C, Riss P, Selberherr A, Koperek O, Niederle B (2020). Early diagnosis of medullary thyroid cancer: are calcitonin stimulation tests still indicated in the era of highly sensitive calcitonin immunoassays?. Thyroid.

[13] Opsahl EM, Akslen LA, Schlichting E, Aas T, Brauckhoff K, Hagen AI (2019). The role of calcitonin in predicting the extent of surgery in medullary thyroid carcinoma: a nationwide population-based study in Norway. Eur Thyroid J.

[14] Yip DT, Hassan M, Pazaitou-Panayiotou K, Ruan DT, Gawande AA, Gaz RD (2011). Preoperative basal calcitonin and tumor stage correlate with postoperative calcitonin normalization in patients undergoing initial surgical management of medullary thyroid carcinoma. Surgery.

[15] Patel KN, Yip L, Lubitz CC, Grubbs EG, Miller BS, Shen W (2020). The American association of endocrine surgeons guidelines for the definitive surgical management of thyroid disease in adults. Ann Surg.

[16] Perros P, Boelaert K, Colley S, Evans C, Evans RM, Gerrard Ba G (2014). Guidelines for the management of thyroid cancer. Clin Endocrinol (Oxf).

[17] Dralle H, Machens A (2013). Surgical management of the lateral neck compartment for metastatic thyroid cancer. Curr Opin Oncol.

[18] McMullen C, Rocke D, Freeman J (2017). Complications of bilateral neck dissection in thyroid cancer from a single high-volume center. JAMA Otolaryngol Head Neck Surg.

[19] Scollo C, Baudin E, Travagli JP, Caillou B, Bellon N, Leboulleux S (2003). Rationale for central and bilateral lymph node dissection in sporadic and hereditary medullary thyroid cancer. J Clin Endocrinol Metab.

[20] Polistena A, Monacelli M, Lucchini R, Triola R, Conti C, Avenia S (2015). Surgical morbidity of cervical lymphadenectomy for thyroid cancer: a retrospective cohort study over 25 years. Int J Surg.

[21] Amin MB, Greene FL, Edge SB, Compton CC, Gershenwald JE, Brookland RK (2017). The Eighth Edition AJCC cancer staging manual: continuing to build a bridge from a population-based to a more “personalized” approach to cancer staging. CA Cancer J Clin.

[22] Moreira VF, Garrido E (2014) Información Al Paciente ¿Qué Es El Absceso Hepático? Rev esp enfeRm dig 106(5):2014. Available from: http://scielo.isciii.es/pdf/diges/v106n5/paciente.pdf

[23] Schlumberger M, Bastholt L, Dralle H, Jarzab B, Pacini F, Smit JWA (2012). 2012 European thyroid association guidelines for metastatic medullary thyroid cancer. Eur Thyroid J.

[24] Park H, Park J, Choi MS, Kim J, Kim H, Shin JH (2020). Preoperative serum calcitonin and its correlation with extent of lymph node metastasis in medullary thyroid carcinoma. Cancers (Basel).

[25] Polistena A, Sanguinetti A, Lucchini R, Galasse S, Monacelli M, Avenia S (2017). Timing and extension of lymphadenectomy in medullary thyroid carcinoma: a case series from a single institution. Int J Surg.

[26] Frank-Raue K, Raue F (2015). Hereditary medullary thyroid cancer genotype-phenotype correlation. Recent Results Cancer Res Fortschritte der Krebsforsch Prog dans les Rech sur le Cancer.

[27] Haddad RI, Nasr C, Bischoff L, Busaidy NL, Byrd D, Callender G (2018). Thyroid carcinoma, version 2.2018 featured updates to the NCCN guidelines. JNCCN J Natl Compr Cancer Netw.

[28] Machens A, Lorenz K, Dralle H (2009). Individualization of lymph node dissection in RET (rearranged during transfection) carriers at risk for medullary thyroid cancer: value of pretherapeutic calcitonin levels. Ann Surg.

[29] Hwang HS, Orloff LA (2011). Efficacy of preoperative neck ultrasound in the detection of cervical lymph node metastasis from thyroid cancer. Laryngoscope.

[30] Zhu Q, Shao Z, Zhang X, Xu D (2021). Correlation between ultrasonic features of medullary thyroid carcinoma and cervical lymph node metastasis. Ultrasound Med Biol.

